# Audiovisual correspondence between musical timbre and visual shapes

**DOI:** 10.3389/fnhum.2014.00352

**Published:** 2014-05-30

**Authors:** Mohammad Adeli, Jean Rouat, Stéphane Molotchnikoff

**Affiliations:** ^1^Neurocomputational and Intelligent Signal Processing Research Group (NECOTIS), Département de Génie Électrique et de Génie Informatique, Faculté de Génie, Université de SherbrookeSherbrooke, QC, Canada; ^2^Neuroscience Lab, Département de Sciences Biologiques, Université de MontréalMontréal, QC, Canada

**Keywords:** sensory substitution, audiovisual correspondences, timbre, pitch, shapes, color, synesthesia

## Abstract

This article investigates the cross-modal correspondences between musical timbre and shapes. Previously, such features as pitch, loudness, light intensity, visual size, and color characteristics have mostly been used in studies of audio-visual correspondences. Moreover, in most studies, simple stimuli e.g., simple tones have been utilized. In this experiment, 23 musical sounds varying in fundamental frequency and timbre but fixed in loudness were used. Each sound was presented once against colored shapes and once against grayscale shapes. Subjects had to select the visual equivalent of a given sound i.e., its shape, color (or grayscale) and vertical position. This scenario permitted studying the associations between normalized timbre and visual shapes as well as some of the previous findings for more complex stimuli. One hundred and nineteen subjects (31 females and 88 males) participated in the online experiment. Subjects included 36 claimed professional musicians, 47 claimed amateur musicians, and 36 claimed non-musicians. Thirty-one subjects have also claimed to have synesthesia-like experiences. A strong association between timbre of envelope normalized sounds and visual shapes was observed. Subjects have strongly associated soft timbres with blue, green or light gray rounded shapes, harsh timbres with red, yellow or dark gray sharp angular shapes and timbres having elements of softness and harshness together with a mixture of the two previous shapes. Color or grayscale had no effect on timbre-shape associations. Fundamental frequency was not associated with height, grayscale or color. The significant correspondence between timbre and shape revealed by the present work allows designing substitution systems which might help the blind to perceive shapes through timbre.

## 1. Introduction

The aim of this work is to investigate correspondences between musical timbre and shapes. This investigation rests on the assumption that there are perceptual correspondences between auditory and visual modalities. This assumption is supported by the common properties of sound and light (Caivano, 1994) and studies of synesthesia which have found evidence of these correspondences. Synesthesia is a condition in which physical stimulation of a sensory system gives rise to automatic and involuntary perception of a different stimulus modality (Cytowic, 1995; Heer, 2000; Hubbard, 2007; Cytowic and Eagleman, 2009). Those who experience synesthesia are called synesthetes. Experiments have revealed that non-synesthetes can similarly match auditory features (e.g., pitch and loudness) with visual features (e.g., light intensity, size and elevation). For instance, non-synesthetes have been able to associate auditory loudness with light intensity (louder auditory tones with higher light intensities) (Marks, 1974, 1987), color saturation (louder auditory tones with higher color saturations) (Giannakis and Smith, 2001); auditory pitch with visual shape (lower auditory pitches with curvy shapes and higher auditory pitches with sharp angular shapes) (Melara and O'Brien, 1987), visual size (higher auditory pitches with smaller sizes (Walker and Smith, 1985; Mondloch and Maurer, 2004; Parise and Spence, 2008, 2009; Evans and Treisman, 2010; Parise and Spence, 2012), light intensity (higher auditory pitches with higher light intensities) (Bernstein and Edelstein, 1971; Marks, 1974, 1987; Melara, 1989; Hubbard, 1996; Collier and Hubbard, 2001; Marks, 2004), elevation (higher auditory pitches with higher visual elevations) (Melara and O'Brien, 1987; Ben-Artzi and Marks, 1995; Patching and Quinlan, 2002; Gallace and Spence, 2006; Rusconi et al., 2006; Eitan and Timmers, 2010; Evans and Treisman, 2010; Chiou and Rich, 2012).

Unfortunately only few researchers have studied the relation between timbre and visual cues. In some experiments not aimed at studying timbre directly, subjects selected a curvy shape for nonsense word “Baluba” or “Bouba” and a sharp jagged shape for nonsense word “Takete” or “Kiki” (Köhler, 1947; Ramachandran and Hubbard, 2001; Maurer et al., 2006; Parise and Spence, 2012). In addition, in Parise and Spence (2012), sine waves were also associated with the curvy shape while square waves were associated with the jagged one. In another study, participants reliably correlated visual texture contrast with sound brightness, visual texture periodicity with auditory dissonance, and visual texture coarseness and granularity with sound compactness (Giannakis, 2006b). In Fernay et al. (2012), a synesthete and 10 control subjects were asked to draw a shape for each sound and determine two colors as well as a vertical and a horizontal position for it. Sounds included four English vowels which had been manipulated in Praat software (Boersma and van Heuven, 2001). For both the synesthete and control subjects, vertical position and color brightness increased with auditory pitch and the speaker's gender determined the size of the shape (larger shapes for male voice). However, synesthete's responses were more consistent over time (Fernay et al., 2012).

The audio–visual correspondences can play a key role in the development of more efficient auditory–visual substitution systems and interactive musical interfaces. Some people including Louis-Bertrand Castel have designed ocular harpsichords or color organs to play color music (Franssen, 1991; Peacock, 1988; Orlandatou, 2009). Abbado created an audio–visual piece of music called “Dynamics” in which he had made good use of musical timbre to enhance the process of composition (Abbado, 1988). Some applications which have taken advantage of audio–visual correspondences include Sound Mosaics designed for sound synthesis (Giannakis, 2006a), an intuitive interactive interface for visual representation of sounds (Grill and Flexer, 2012) and a 3D immersive system for representation and manipulation of musical processes (Berthaut et al., 2010, 2011).

While reviewed studies have revealed non-synesthetes' ability to make audio–visual associations and found some correspondences between auditory and visual features, auditory stimuli in most of these studies have been limited to simple synthetic tones. Considering the less salient features of complex sounds which might not be attended at all, the question then arises: are these correspondences also valid for more complex sounds (e.g., music)? More importantly, hypothetically possible associations between shapes and timbres, which are sophisticated features of visual objects and sounds, have not received enough attention yet and therefore should be subject of more investigation. To address these issues, we conducted an experiment in which the windowed sounds of eight musical instruments equalized in energy were used as auditory stimuli. This allowed us to investigate associations between two important features of music i.e., pitch and normalized timbre and visual features i.e., shapes, colors, grayscales and height (vertical position of a visual stimulus above a given level). The objectives of the experiment were multifold. First and foremost, we could study the potential associations between timbre and visual shapes and observe how they might be affected by auditory pitch and color (gray scale) if such associations indeed existed. Secondly, we could examine some of the previous findings, i.e., associations between pitch and elevation, pitch and color intensity, pitch and hue in the context of more complex auditory stimuli. Another important goal of this investigation was to design the experiment protocol in such a way that results could be quantifiable and applicable in an auditory visual substitution system.

## 2. Materials and methods

### 2.1. Subjects

One hundred and nineteen subjects (31 females and 88 males) participated in the experiment. All subjects did the experiments online (http://audio-visual-experiment.espaceweb.usherbrooke.ca). In the beginning they had to fill in a questionnaire including questions about their synesthetic experiences (if any), music training background, age, sex and native language. According to the information provided by the subjects, they were from 19 to 63 years old (mean = 30, SD = 10). Thirty-one subjects have claimed to see things which are not there when they hear sounds or vice versa. This can be considered a kind of synesthesia-like or audio–visual experience but should not be generalized to synesthesia due to lack of evidence. Thirty-six subjects had little or no music training, 47 were amateur musicians and 36 were professionals. All subjects reported that they had normal (or corrected to normal) vision and hearing. On average the experiment lasted 8.2 min (SD = 2.8). The subjects, who were mostly from academia, participated in the experiment voluntarily and were not in contact with authors.

### 2.2. Auditory and visual stimuli

**Figure 1:**
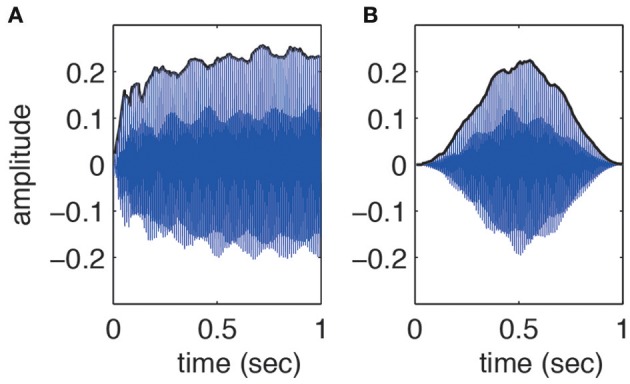
**The waveform of the sound derived from Cello with fundamental frequency of 100 Hz (A) before and (B) after having been manipulated with a Hanning window**. Hanning window is a bell-shaped weighting function which makes the sound's onset and offset symmetrical. However, the harmonic structure of the sound is preserved.

This research utilized RWC Music Database (Musical Instrument Sound) (Goto, 2004; Goto et al., 2003). All sounds in this database are sampled at 44.1 KHz. We selected 8 musical instruments i.e., Electric Piano, Marimba, Classic Guitar, Cello, Tenor Sax, Triangle, crash Cymbals and Gong (RWC-MDB-I-2001 No. 02, No. 05, No. 09, No. 17, No. 27, No. 41, No. 41, No. 44). For the first five instruments, four different fundamental frequencies (100, 150, 200, 250 Hz) were selected and used. One-second segment duration of these sounds was windowed by a Hanning window (in MATLAB) such that the sounds produced by five different instruments still shared the same normalized temporal envelope (see Figure 1 for an example). Thus listeners could not make their decision based on the true onsets and offsets of the sounds i.e., they could not use the full range of musical timbre, making the distinction more difficult. However, sounds of percussive instruments i.e., Triangle, Crash Cymbals and Gong were not windowed. So the total number of sounds was 3 instruments + 5 instruments * 4 fundamentals = 23. The auditory stimuli were all equalized in energy and randomly presented twice to the subjects.

**Figure 2:**
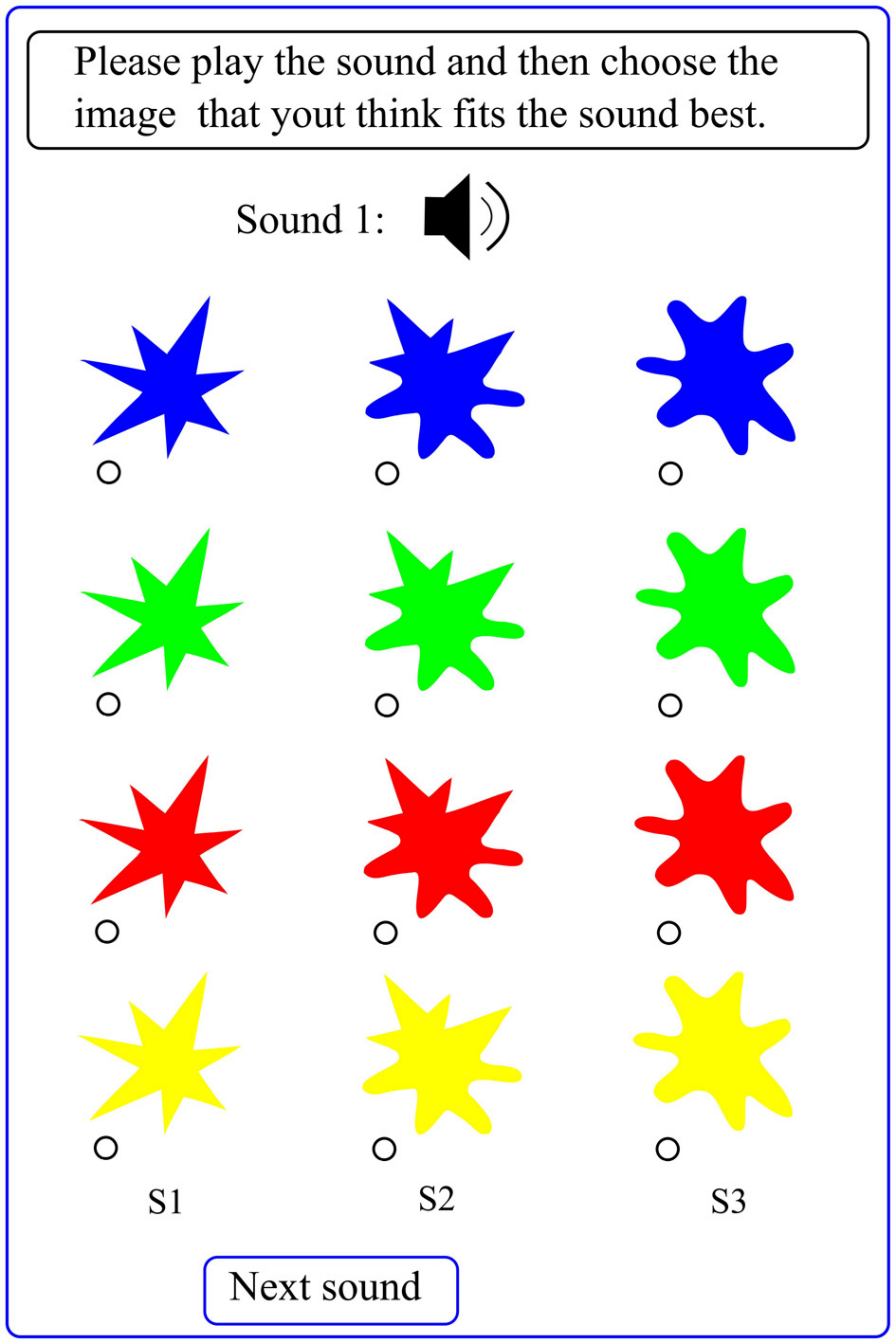
**The web interface designed for the experiment: each page includes 12 visual stimuli i.e., 3 shapes by either four colors (blue, green, red, and yellow) or four grayscales (0 (black), 0.3, 0.6, and 0.9)**. Each row has a different color (grayscale) which changes randomly. Subjects had to play the sound and select its visual equivalent. Whenever needed in this article, labels S1, S2, and S3 are used to refer to the three shapes.

Visual stimuli included 12 colored or grayscale shapes, that is, three shapes which appeared in four colors (blue, green, red, and yellow) or in four grayscales (0 (black), 0.3, 0.6, and 0.9 (lightest scale)) on the screen (see Figure 2 for an example). On each page, shapes were either colored or grayscale. Each row of shapes had a different color (or grayscale) but this color (or grayscale) varied randomly across pages. Colored and grayscale pages were also presented in randomized order. Each sound was presented once against colored shapes (blue–green–red–yellow) and once against grayscale shapes. The reason for using both color and grayscale was to observe their effect on potential correspondences between timbre and shapes. Thus, the auditory stimuli varied in two features i.e., timbre and fundamental frequency while visual stimuli varied in shape, color, grayscale and height (each row of shapes had a different height).

### 2.3. Procedure

A web interface was designed to run the experiment. A typical page of this interface is shown in Figure 2. All subjects did the experiment online. No training was provided to the subjects to make the experiment more spontaneous and natural. They had no prior information about the experiment, the auditory and visual stimuli, and the goals of the experiment. First they were required to provide some personal information. Next, on each page, they were randomly presented with 12 visual stimuli and one auditory stimulus. Subjects had to play the sound (by pressing a button) and then choose a visual stimulus that they thought fitted the sound best. There were 46 auditory stimuli (23 sounds each of which was presented twice) for which subjects had to determine their equivalent visual representation.

As stated in section 2.1, there existed a great variability among subjects in terms of gender, music training and synesthesia-like experiences. The last two factors were judged by the subjects themselves and thus we had no evidence to what extent these judgments had been objective. Therefore, instead of considering different groups of subjects (e.g., musicians, non-musicians, synesthetes, non-synesthetes, males and females), a clustering algorithm was used to find potential groups of subjects based on the similarity of their responses. This algorithm was *Density-based spatial clustering of applications with noise (DBSCAN)* (Ester et al., 1996) which is described in next section. In addition, to investigate the homogeneity of shape selections across age groups, three groups were selected and studied. The results are presented in section 3.2.

### 2.4. Clustering using DBSCAN

The goal of the clustering was to search for potential groups of subjects who might have followed a similar strategy in the choice of shapes for all sounds i.e., for which the within group distance was smaller than the between group distances. A measure of distance between subjects *i* and *j* was defined as:

(1) $$D_{i,j}=\sum_{n\;=\;0}^{N}|S_{i}(n)-S_{j}(n){|}^{2}$$

Where *N* was 46 (twice the number of sounds) and *S_i_*(*n*) was the shape selected for sound *n* by subject *i*. If subjects *i* and *j* had chosen the same shape for sound *n*, |*S_i_*(*n*)−*S_j_*(*n*)| would have been set equal to zero and otherwise to 1. Consequently the distance measure varied from 0 (all responses exactly the same) to 46 (all responses different).

DBSCAN required, in the first place, the minimum number of subjects *min*_*num* of a cluster and the radius ε of a neighborhood to be determined (Ester et al., 1996). Then subject *k* was chosen randomly. If the number of people in the subject's “ε − *neighborhood*” (a neighborhood of radius ε centered on the subject) was less than *min*_*num*, the subject was considered as noise otherwise a cluster was built. In the next step, DBSCAN tried to extend the found cluster by examining the cluster members. For each cluster member, an ε − *neighborhood* was built and if the number of subjects in it was greater than or equal to *min*_*num*, all members of this neighborhood were added to the cluster. This process continued until all subjects were examined.

In our analysis, *min*_*num* and ε were 5 and 7, respectively. Therefore, to find a cluster, there had to be at least five subjects in it and subjects belonging to an ε − neighborhood of this cluster could have at most seven different answers (~15%).

### 2.5. Statistical analyses

Fisher's exact tests have been conducted in section 3.2 to test the hypothesis of no association between age and shape selection strategy. In sections 3.3, 3.5, 3.6, 3.7, and 3.9, Chi-squared goodness of fit tests have been used to examine whether shape, color, grayscale and height selections have arisen by chance. In section 3.4, Stuart-Maxwell's Chi-squared test have been performed to investigate the marginal homogeneity of colored and grayscale shape selections at a given fundamental frequency. In section 3.8, McNemar's Chi-squared tests have been used to examine if the distribution of dark and light grayscale selections were independent of fundamental frequency. In sections 3.4, 3.6, and 3.8, the confidence interval for the difference between proportions *P*_1_ and *P*_2_ from a multinomial distribution have been calculated by:

(2) $$DIFF(P_{1},\;P_{2})=P1-P2\pm Z_{\alpha }\sqrt{\frac{P_{1}+P_{2}-{\left(P_{1}-P_{2}\right)}^{2}}{N}}$$

Where *N* (number of subjects) is 119, α is Bonferroni adjusted significance level and *Z*_α_ is its corresponding *Z*-score. The experiment wide significance level α is 0.05 while in a set of m tests, significance level (per test) α is equal to $\frac{\bar{\alpha }}{m}$.

## 3. Results

### 3.1. Clusters found by DBSCAN

DBSCAN found only one cluster of 103 subjects and considered 16 subjects as noise. These 16 subjects were in no other way different from the found cluster, that is to say, the distributions of females, males, musicians, non-musicians, synesthetes and non-synesthetes among them were approximately the same as those of the found cluster. The only certain thing about them is that their answers were very different from the found cluster. The fact that only one cluster was found implies that gender, music training and claimed synesthetic experiences have not played a significant role in audio–visual associations in our experiment. For the rest of this paper, the results for all subjects (119) are presented.

### 3.2. Shape selections across age groups

To examine the effect of age on shape selections, subjects were divided into three groups. First group consisted of 79 subjects (19–30 years old) while the second and third groups consisted of 29 subjects (31–45 years old) and 11 subjects (46–63 years old), respectively. In order to compare a group with another, 23 Fisher's exact tests were performed (one per sound). To achieve an experiment-wide significance level of α = 0.05, Bonferroni adjusted significance level of $\alpha =\frac{\bar{\alpha }}{23}=0.00217$ was used per test. No significant difference between these age groups was found. For all sounds *p* ≥ 0.004, χ^2^ < 9.83. This suggests that age has not had an effect on shape selections.

### 3.3. The relationship between timbre and shapes

Chi-squared goodness of fit tests were conducted to determine whether shapes had been selected randomly for the 23 stimuli. Bonferroni adjusted significance level of $\alpha =\frac{0.05}{23}=0.00217$ was used per test (as explained above). Shape selection was very unlikely to have arisen by chance: for all sounds χ^2^(2, *N* = 119) ≥ 32.89, *p* < 0.000001; for instance for Chello at 100 Hz, χ^2^(2, *N* = 119) = 53.76, *p* < 0.000001 and for Piano at 250 Hz, χ^2^(2, *N* = 119) = 41.16, *p* < 0.000001. Shape selection strategy has therefore differed with timbre. Table 1 shows the mostly selected shapes for all timbres. In this table, CMC (Confidence Measure for Colored shapes) and CMG (Confidence Measure for Grayscale shapes) show what percentage of subjects have agreed on the selected shape. For instance, a rounded shape has been associated with the sound derived from Piano but this association is stronger for grayscale shapes (CMG = 84%) than for colored shapes (CMC = 64%). CMC and CMG percentages are much higher than chance level (33%) which implies that the association between timbre and shapes is strong.

**Table 1:** **Shape selections for timbre: the colored and grayscale shapes selected for each sound are shown in this table**.

**Instrument**	**Fundamental frequency F0 (Hz)**														
	**100**			**150**			**200**			**250**			**No F0**		
	**Shape**	**CMC (%)**	**CMG (%)**	**Shape**	**CMC (%)**	**CMG (%)**	**Shape**	**CMC (%)**	**CMG (%)**	**Shape**	**CMC (%)**	**CMG (%)**	**Shape**	**CMC (%)**	**CMG (%)**
Cello	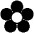	50	52	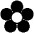	77	63	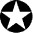	63	58	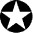	61	62	–	–	–
Guitar	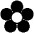	64	61	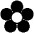	76	73	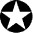	55	51	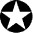	55	53	–	–	–
Piano	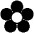	61	79	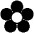	73	73	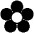	71	71	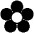	50	51	–	–	–
Marimba	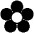	56	53	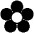	82	71	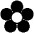	81	76	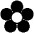	75	71	–	–	–
Sax	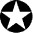	59	59	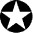	72	66	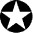	69	64	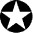	62	70	–	–	–
Triangle	–	–	–	–	–	–	–	–	–	–	–	–	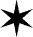	89	94
Carsh cymbals	–	–	–	–	–	–	–	–	–	–	–	–	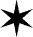	92	85
Gong	–	–	–	–	–	–	–	–	–	–	–	–	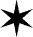	73	87

CMC and CMG are two confidence measures which show what percentages of subjects agreed on the selected shape in cases of colored and gray scale shapes. Symbols 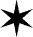, 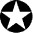, and 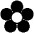 represent shapes S1, S2, and S3 in Figure 2. Shape selection strategy has changed with timbre (see statistics in text). Chance level is 33%. Sounds of the first five instruments were pre-processed by a Hanning window.

### 3.4. Does shape selection depend on fundamental frequency?

Table 1 shows that the shapes selected for sounds derived from Cello and Guitar have changed with fundamental frequency. For lower fundamental frequencies (100 and 150 Hz) these sounds had rounded shapes but for higher fundamentals (200 and 250 Hz) they had partly rounded partly angular shapes. Table 1 shows no change in associations between shapes and fundamental frequency for sounds derived from Piano, Marimba and Sax. However, to better study the shape-fundamental frequency association, all the shapes had to be taken into account not just the mostly selected one for a given sound. To compute the shape-fundamental frequency association, timbre differences were ignored i.e., the timbre-shape associations were averaged over sounds derived from five instruments (Cello, Guitar, Piano, Marimba, and Sax) by:

(3) $$\begin{array}{l} P\text{​}\left(S_{k}|F_{i}\right)=\sum_{j\;=\;1}^{5}P\text{​}\left(S_{k},\;T_{j}|F_{i}\right)=\sum_{j\;=\;1}^{5}P\text{​}\left(S_{k}|T_{j},\;F_{i}\right)P\text{​}\left(T_{j}|F_{i}\right)\\ \;=\sum_{j\;=\;1}^{5}P\text{​}\left(S_{k}|T_{j},\;F_{i}\right)\frac{P\text{​}\left(F_{i}|T_{j}\right)P\text{​}\left(T_{j}\right)}{P\text{​}\left(F_{i}\right)}\\ \;=\sum_{j\;=\;1}^{5}P\text{​}\left(S_{k}|T_{j},\;F_{i}\right)\frac{\left(0.25\right)\left(0.2\right)}{\left(0.25\right)}\\ \;=0.2\sum_{j\;=\;1}^{5}P\text{​}\left(S_{k}|T_{j},\;F_{i}\right) \end{array}$$

**Figure 3:**
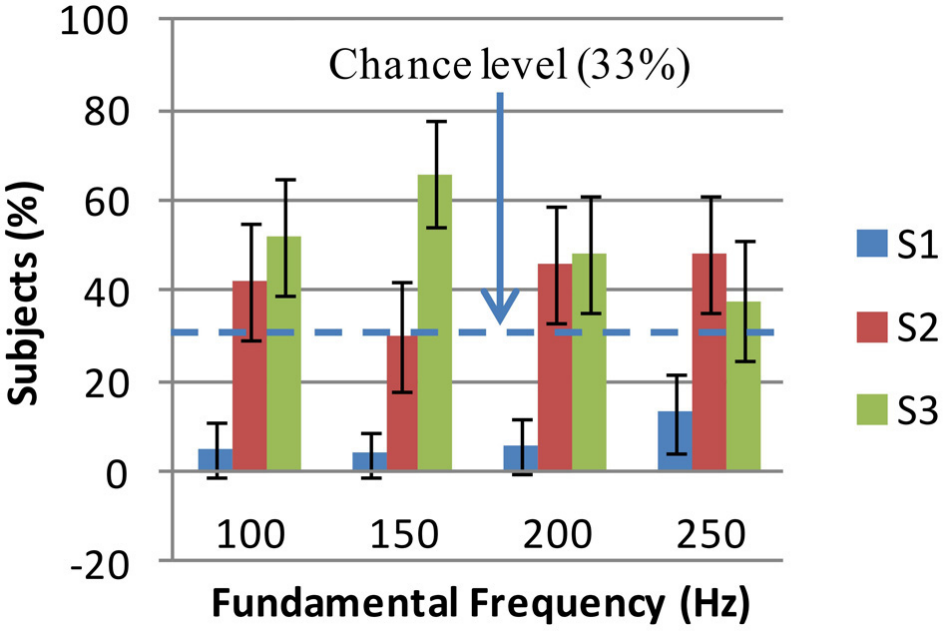
**Colored shape selections for fundamental frequency: the percentages of subjects who have selected shapes S1 (jagged), S2 (mixed), and S3 (rounded) for each fundamental frequency are shown**. The error bars show Bonferroni adjusted 99.59% confidence intervals (obtained by *Z*-test). S1 is the least selected shape at all frequencies (<13%). There is no significant difference between S3 and S2 percentages except at 150 Hz where S3 is the mostly selected shape (66%). At other frequencies, S2 and S3 are almost equally selected (see text for details). S1, S2, and S3 are the shapes shown in Figure 2.

**Figure 4:**
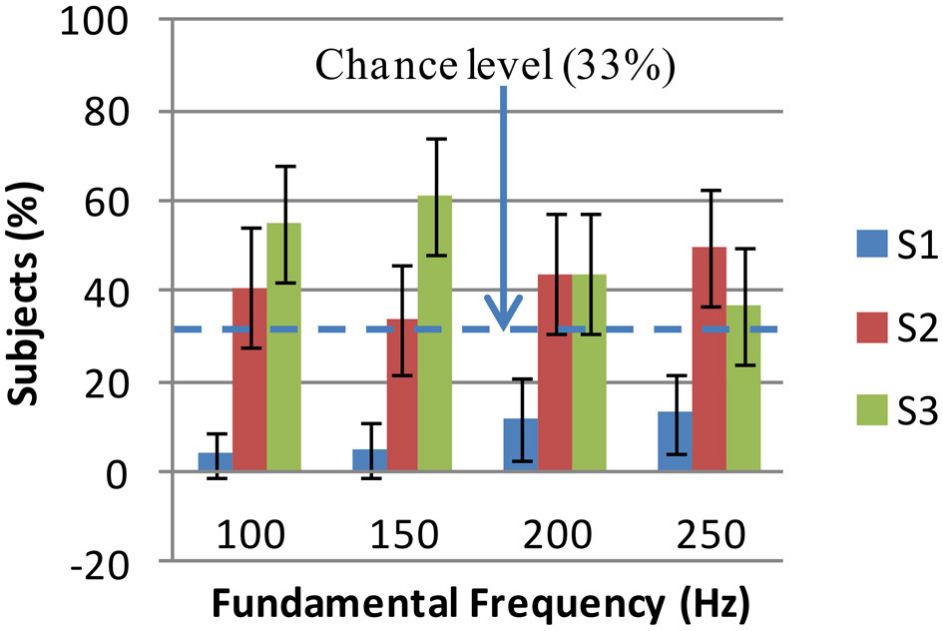
**Grayscale shape selections for fundamental frequency: the percentages of subjects who have selected shapes S1 (jagged), S2 (mixed), and S3 (rounded) for each fundamental frequency are shown**. The error bars show Bonferroni adjusted 99.59% confidence intervals (obtained by *Z*-test). S1 is the least selected shape at all frequencies (< 13%). There is no significant difference between S3 and S2 percentages except at 150 Hz where S3 is the mostly selected shape (61%). At other frequencies, S2 and S3 are almost equally selected (see text for details). S1, S2, and S3 are the shapes shown in Figure 2.

Where *F*, *T*, and *S* represent fundamental frequency, timbre and shape, respectively; *i* = 1 : 4, *j* = 1 : 5, and *k* = 1 : 3; *P*(.) is the probability measure; *P*(*F_i_*) = $\frac{1}{4}$, *P*(*T_j_*) = $\frac{1}{5}$, *P*(*F_i_*|*T_j_*) = $\frac{1}{4}$; and 100 * *P*(*S_k_*|*T_j_*, *F_i_*) is the percentage of subjects who have selected the shape *S_k_* for an auditory stimulus of timbre *T_j_* and frequency *F_j_*. This distribution was known from the results of the experiments. The shape-fundamental frequency associations *P*(*S_k_*|*F_i_*) were computed for two cases: colored shapes and grayscale shapes. These associations are shown in Figures 3, 4. Using Stuart–Maxwell's test for marginal homogeneity, no significant difference was observed between marginal distributions of colored shapes in Figure 3 and respective marginal distributions of grayscale shapes in Figure 4: at 100 Hz, (Stuart–Maxwell's χ^2^(2, *N* = 119) = 0.3022, *p* = 0.8598); at 150 Hz, (Stuart–Maxwell's χ^2^(2, *N* = 119) = 0.5904, *p* = 0.7444); at 200 Hz, (Stuart–Maxwell's χ^2^(2, *N* = 119) = 3.5960, *p* = 0.1656); and at 250 Hz, (Stuart–Maxwell's χ^2^(2, *N* = 119) = 0.0251, *p* = 0.9875). Bonferroni adjusted significance level was $\alpha =\frac{0.05}{4}=0.0125$ per test. This suggests that color and gray scale have not had a significant effect on the associations between shapes and fundamental frequency.

In Figure 3 (colored shape selection for fundamental frequency), a set of 12 *Z*-tests (three tests per frequency) were performed to examine the differences between S1, S2, and S3 selections. Boneferroni adjusted significance level of $\alpha =\frac{0.05}{12}=0.0041$ (corresponding to a two-tailed *Z*_α_ of 2.87 and a confidence level of 99.59% per test) was considered (see section 2.5 for details of the test). In this figure, significant differences between S2 (mixed) and S1 (jagged) shapes selections were observed at all frequencies. These differences lay in intervals [22%, 52%] (at 100 Hz), [12%, 40%] (at 150 Hz), [24%, 56%] (at 200 Hz), and [17%, 53%] (at 250 Hz) with 99.59% confidence. The difference between S3 (rounded) and S2 (mixed) selections was only significant at 150 Hz and fell in interval [12%, 60%] with 99.59% confidence.

In Figure 4 (grayscale shape selections for fundamental frequency), significant differences between S2 (mixed) and S1 (jagged) selections were found at all frequencies using another set of 12 *Z*-tests similar to above-mentioned ones. These differences lay in intervals [22%, 52%] (100 Hz), [14%, 44%] (150 Hz), [14%, 50%] (200 Hz), and [19%, 55%] (250 Hz) with 99.59% confidence. However, like in Figure 3, the difference between S3 (rounded) and S2 (mixed) selections was only significant at 150 Hz and was in interval [2%, 52%] with 99.59% confidence. In general, effects of fundamental frequency on shape selections are such that at 150 Hz, the rounded (S3) shape has been mostly selected; at 100, 200, and 250 Hz the rounded (S3) and mixed (S2) shapes have been selected almost equally while the jagged shape (S1) has been the least selected one at all fundamental frequencies.

### 3.5. Relationship between timbre and color

Mostly selected colors for different timbres are presented in Table 2. In this table, CMC is a confidence measure which shows what percentage of subjects have agreed on the color selected for a given timbre. Chance level is 25% in this table. Chi-squared goodness of fit tests were also performed to determine whether colors had been selected randomly for the 23 stimuli. Bonferroni adjusted significance level of $\alpha =\frac{0.05}{23}=0.00217$ was used per test. Color selections for the following five sounds were not significantly different from chance level: sounds derived from Guitar at 150 Hz, (χ^2^(3, *N* = 119) = 14.24, *p* = 0.0026); Marimba at 200 Hz, (χ^2^(3, *N* = 119) = 14.41, *p* = 0.0024); Sax at 150 Hz, (χ^2^(3, *N* = 119) = 4.13, *p* = 0.248); Sax at 200 Hz, (χ^2^(3, *N* = 119) = 4.06, *p* = 0.255); Sax at 250 Hz, (χ^2^(3, *N* = 119) = 7.23, *p* = 0.065). With regard to the other 18 sounds, Table 2 suggests that sounds with rounded shapes (according to Table 1) have mostly been associated with blue and green while sounds with sharp angular or mixed shapes have been associated with yellow and red. For instance, sounds derived from Piano and Marimba which have soft timbres (and rounded shapes according to Table 1) have been associated with blue and green. However, sounds of Crash Cymbals, Gong and Triangle which have harsh timbres (and sharp angular shapes) have been matched with red or yellow. The fact that CMC levels were at chance level for five sounds and low for some others implies that the timbre-color associations are weaker than timbre-shape associations.

**Table 2:**
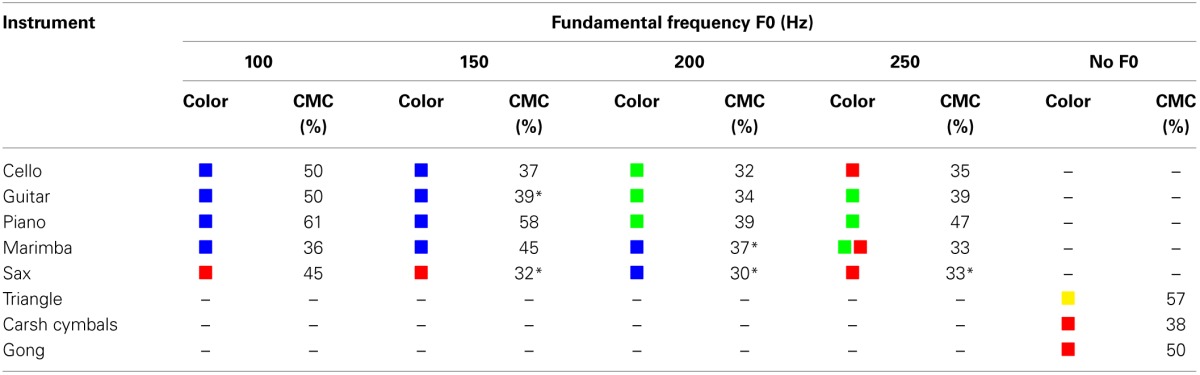
**Color selections for timbre: this table shows the color selected for each sound**.

CMC is a confidence measure which shows what percentage of subjects agreed on the selected color. Sounds derived from Cello, Guitar, Marimba, and Piano have mostly been associated with blue and green while those of Sax, Triangle, Crash Cymbals, and Gong have mostly been associated with red and yellow. The chance level is 25%. The distribution of colors for sounds marked with asterisks were not different from chance level even though two of them have relatively high CMC levels (see text for statistics).

### 3.6. Does color selection depend on fundamental frequency?

To formulate the color-fundamental frequency associations, timbre differences were ignored i.e., the timbre-color associations were averaged over sounds derived from five instruments (Cello, Guitar, Piano, Marimba, and Sax) by:

(4) $$\begin{array}{l} P\text{​}\left(C_{k}|F_{i}\right)=\sum_{j\;=\;1}^{5}P\text{​}\left(C_{k},\;T_{j}|F_{i}\right)=\sum_{j\;=\;1}^{5}P\text{​}\left(C_{k}|T_{j},\;F_{i}\right)P\text{​}\left(T_{j}|F_{i}\right)\\ \;=\sum_{j\;=\;1}^{5}P\text{​}\left(C_{k}|T_{j},\;F_{i}\right)\frac{P\text{​}\left(F_{i}|T_{j}\right)P(T_{j})}{P(F_{i})}\\ \;=\sum_{j\;=\;1}^{5}P\text{​}\left(C_{k}|T_{j},\;F_{i}\right)\frac{\left(0.25)(0.2\right)}{\left(0.25\right)}\\ \;=0.2\sum_{j\;=\;1}^{5}P\text{​}\left(C_{k}|T_{j},\;F_{i}\right) \end{array}$$

**Figure 5:**
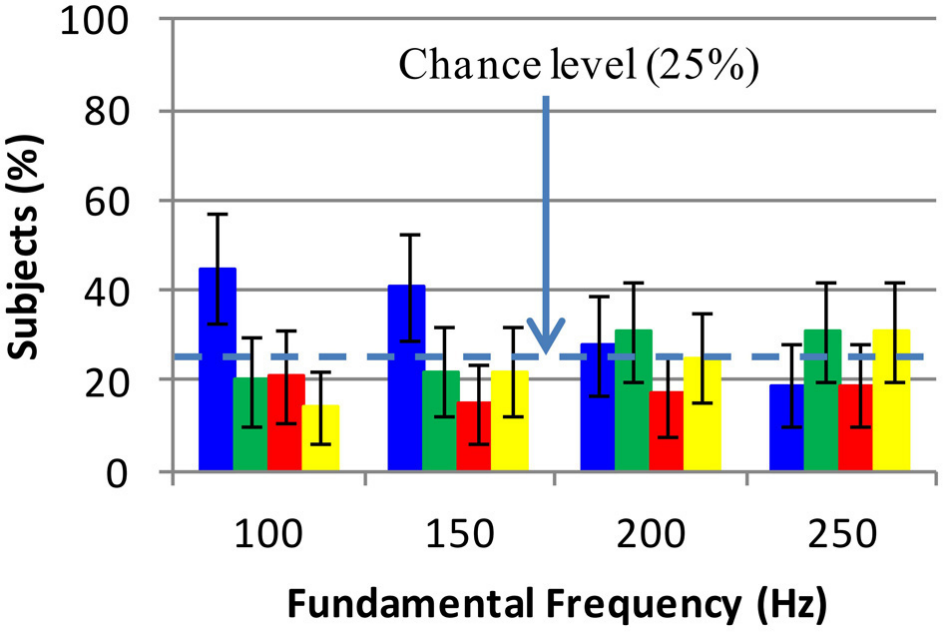
**Color selections for fundamental frequency: each colored bar shows the selection rate of the same color**. The error bars show the 99.17% confidence intervals for the selection rates. Blue has been associated with fundamental frequency 100 Hz. No colors have been associated with 150, 200, and 250 Hz because all selection rates for them are approximately at chance level. Thus the associations between color and fundamental frequency are only strong at 100 Hz.

Where C represents color (*k* = 1 : 4) and the rest of the parameters have been introduced in section 3.4. 100 * *P*(*C_k_*|*T_j_*, *F_i_*) is the percentage of subjects who have selected color *C_k_* for an auditory stimulus of timbre *T_j_* and frequency *F_j_*. This distribution was known from the results of the experiments, e.g., we knew that blue had been mostly selected for Guitar at 100 Hz. The associations between color and fundamental frequency are presented in Figure 5.

Four Chi-squared goodness of fit tests (one per fundamental frequency) were performed to determine whether color selections at each fundamental frequency have been due to chance. Bonferroni adjusted significance level of $\alpha =\frac{0.05}{4}=0.0125$ was used per test. The distribution of colors were significantly different from chance level at 100 and 150 Hz; (χ^2^(3, *N* = 119) = 25.5, *p* < 0.001) and (χ^2^(3, *N* = 119) = 18.04, *p* < 0.001), respectively. At each of these frequencies 6 *Z*-tests were conducted to determine if blue, green, red, and yellow selections were different ($\alpha =\frac{0.05}{6}=0.0083$). At 100 Hz, blue selection rate was significantly different from those of green, red, and yellow. With 99.17% confidence, the differences between blue selections and green, red, and yellow selections lay in intervals [5%, 45%], [5%, 43%], [14%, 48%], respectively at 100 Hz. At 150 Hz, blue selection was only different from red selection. The difference between blue and red selection rates lay in intervals [9%, 43%]. In summary, no strong associations between fundamental frequency and colors except blue (at 100 Hz) were observed. The only strong association is due to the fact that at 100 Hz, Cello, Guitar, Piano, and Marimba have been strongly associated with blue.

### 3.7. Relationship between timbre and grayscale

Table 3 summarizes the associations between timbre and grayscale. In this table, CMG is a confidence measure which shows what percentage of subjects have agreed on the selected grayscale. Chance level is 25% in this table. Based on the results of 23 Chi-squared tests (similar to those explained in section 3.5) color selections for the following seven sounds were not different from chance level with a Bonferroni adjusted significance level of 0.00217: sounds derived from Cello at 250 Hz, (χ^2^(3, *N* = 119) = 1.57, *p* = 0.666); Piano at 100 Hz, (χ^2^(3, *N* = 119) = 2.31, *p* = 0.510); Piano at 150 Hz, (χ^2^(3, *N* = 119) = 9.44, *p* = 0.024); Piano at 250 Hz, (χ^2^(3, *N* = 119) = 11.18, *p* = 0.0108); Marimba at 100 Hz, (χ^2^(3, *N* = 119) = 1.23, *p* = 0.745); Marimba at 150 Hz, (χ^2^(3, *N* = 119) = 14.61, *p* = 0.00218); and Triangle, (χ^2^(3, *N* = 119) = 8.54, *p* = 0.036). With regard to the other 16 sounds, Table 3 suggests that sounds with soft timbres have mostly been associated with lighter grayscales while sounds with harsher timbres have been associated with dark grayscales. For instance, sounds derived from Piano and Marimba at 200 Hz which have soft timbres (and rounded shapes) have been correlated with light grays (0.6 and 0.9) while sounds of Crash Cymbals and Gong which have harsh timbres (and sharp angular shapes) have been correlated with black. Of course there are few exceptions to this rule, for instance the sound derived from Cello at 100 Hz has been matched with black. The fact that grayscale selections for 7 out of 23 sounds were at chance level and that CMG levels for some other sounds are low implies that the timbre-grayscale associations are weaker than timbre-shape associations.

**Table 3:**
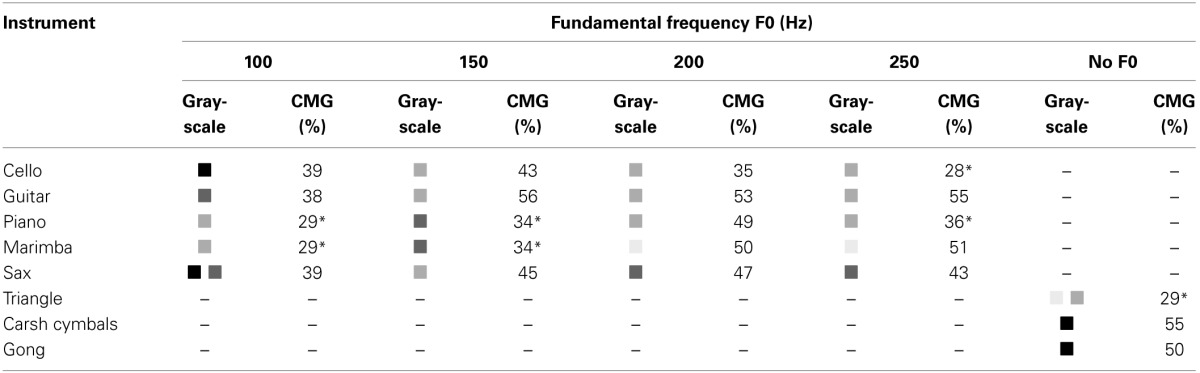
**Grayscale selections for timbre: the grayscale selected for each sound is shown in this table**.

The grayscales are, from the lightest to the darkest, 0.9, 0.6, 0.3, and 0 (black). CMG is a confidence measure which shows what percentage of subjects have agreed on the selected grayscale. Sounds derived from Cello, Guitar, Marimba, and Piano have been mostly associated with light scales (0.6 and 0.9) and those of Sax, Crash Cymbals, and Gong have been mostly associated with dark scales (0 and 0.3). The chance level is 25%. The distribution of grayscales for sounds marked with asterisks were not different from chance level (see text for statistics).

### 3.8. Does grayscale selections depend on fundamental frequency?

To formulate grayscale-fundamental frequency associations, timbre differences were again ignored i.e., the timbre-grayscale associations were averaged over sounds derived from five instruments (Cello, Guitar, Piano, Marimba, and Sax) using equation 4. However, in this equation, colors *C_k_* were replaced with grayscales *G_k_*. The rest of parameters remained unchanged. Thus *P*(*G_k_*|*F_i_*), that is, the association between grayscale and fundamental frequency, was computed using the following equation:

(5) $$P(G_{k}|F_{i})=0.2\sum_{j\;=\;1}^{5}P(G_{k}|T_{j},\;F_{i})$$

**Figure 6:**
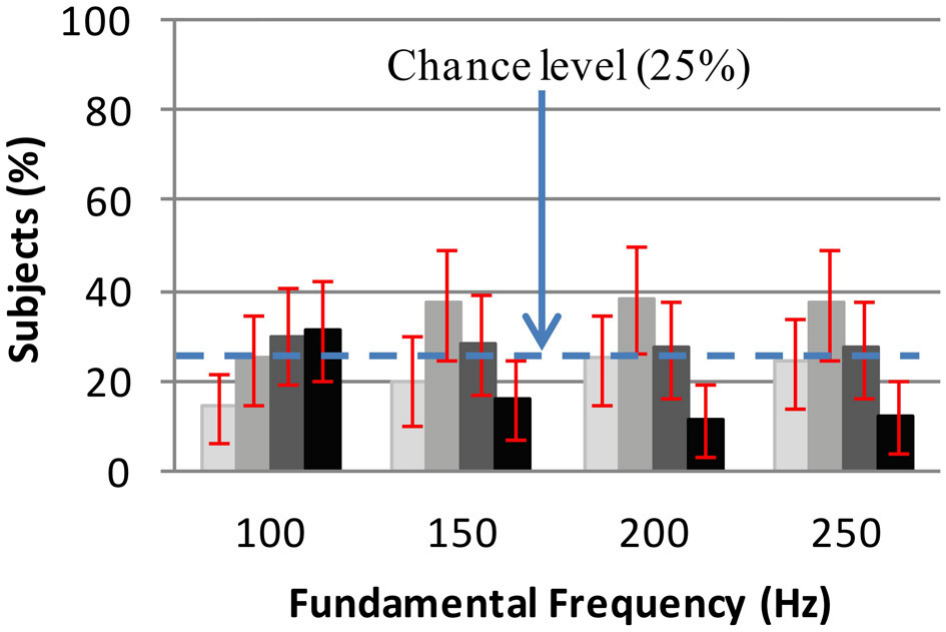
**Grayscale selections for fundamental frequency: each grayscale bar shows the selection rate of the same grayscale**. The error bars show the 98.3% confidence intervals for the selection rates. The grayscales are, from the lightest to the darkest, 0.9, 0.6, 0.3, and 0 (black). When considered alone, the grayscales are not associated with fundamental frequency. But when dark scales (0 and 0.3) are compared with lighter scales (0.6 and 0.9), dark scales selections have decreased while lighter scales selections have increased with fundamental frequency (See details in the text).

The distribution 100 * *P*(*G_k_*|*T_j_*, *F_i_*) which is the percentage of subjects who have selected grayscale *G_k_* for an auditory stimulus of timbre *T_j_* and frequency *F_j_*, was known from the results of the experiments, e.g., we knew that black (grayscale = 0) had mostly been selected for the sound derived from Cello at 100 Hz. The associations between grayscale and fundamental frequency are presented in Figure 6. In this figure, no strong association between fundamental frequency and grayscales was observed due to the lack of a most preferred grayscale at a given fundamental frequency. But when the sum of dark grayscales (0 and 0.3) selections were compared to the sum of lighter grayscales (0.6 and 0.9) distinct patterns were observed. Three McNemar's tests were conducted to determine whether the distribution of light and dark scales had changed from 100 to 150 Hz, from 150 to 200 Hz, and from 200 to 250 Hz. Bonferroni adjusted significance level in this case is $\alpha =\frac{0.05}{3}=0.017$. A significant difference between grayscale distributions at 100 and 150 Hz was observed, (McNemar's χ^2^(1, *N* = 119) = 8.24, *p* < 0.004). The sum of light grayscale selections has increased from 100 to 150 Hz. This increase lay in interval [1%, 35%] with 95% confidence using *Z*-test. Thus low fundamental frequency 100 Hz has been associated with darker scales while higher fundamentals 150, 200, and 250 Hz have been associated with lighter scales.

### 3.9. Relationship between height and fundamental frequency

There were four rows of shapes on each page of our web interface each of which was at a different height (vertical position). Four heights (or elevations) are named *H*_1_, *H*_2_, *H*_3_, and *H*_4_ from the lowest to the highest position. In a similar way to Equation (4), we can show that height and fundamental frequency are related by:

(6) $$P\text{​}\left(H_{m}|F_{i}\right)=0.2\sum_{j\;=\;1}^{5}P\text{​}\left(H_{m}|T_{j},\;F_{i}\right)$$

**Figure 7:**
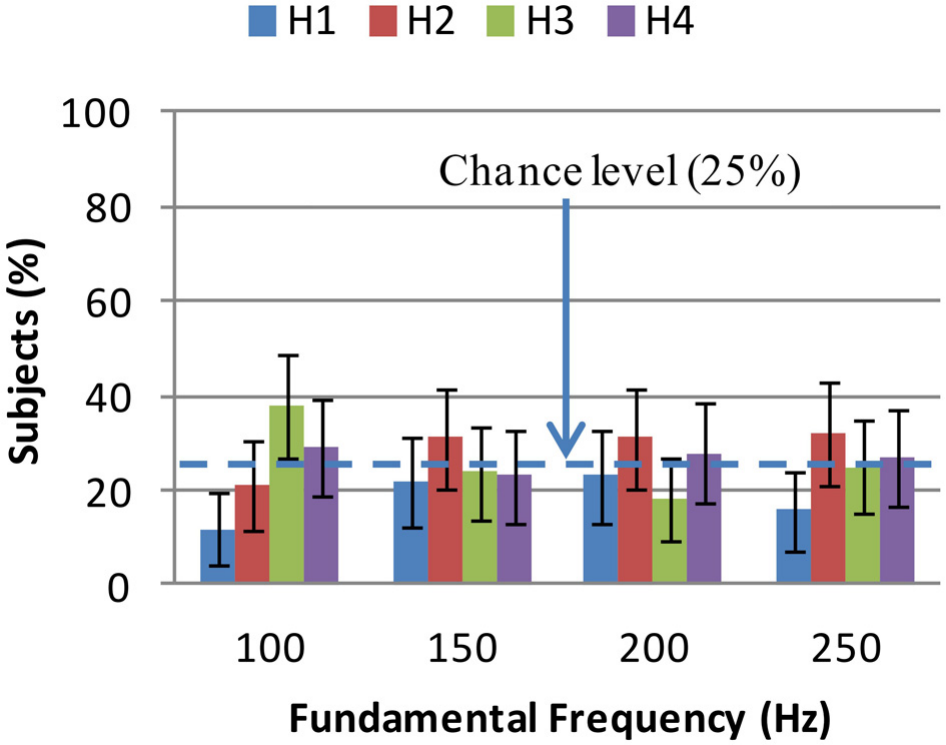
**Height selections for fundamental frequency: H1 is the lowest height and H4 is the highest one**. No organized association between height and fundamental frequency is observed although H3 is the most selected height at 100 Hz. At 150, 200, and 250 Hz, all selection rates are nearly equal to chance level. The error bars show the 98.75% confidence intervals for all selection rates. Chance level is 25%.

100 * *P*(*H_m_*|*T_j_*, *F_i_*) is the percentage of subjects who have chosen the height *H_m_* for an auditory stimulus of timbre *T_j_* and frequency *F_j_*. This distribution was known from the results of the experiments. The distribution *P*(*H_m_*|*F_i_*), that is, the association between height and fundamental frequency is presented in Figure 7. Four Chi-squared goodness of fit tests were conducted to determine whether heights had been selected randomly for each fundamental frequency. Bonferroni adjusted significance level of $\alpha =\frac{0.05}{4}=0.0125$ was used per test. As we can see from Figure 7, for 150, 200, and 250 Hz, all selection rates have been nearly at chance level. Only height selections at 100 Hz were significantly different from chance level (χ^2^(3, *N* = 119) = 17.84, *p* < 0.001). Even for 100 Hz, only the difference between H3 and H1 selection rates was significant *Z* = 4.33, *p* < 0.0001. Despite that, no organized association between fundamental frequency and height was observed.

## 4. Discussion

Results revealed a strong correspondence between timbre and shape such that sounds with harsh timbres (e.g., sounds derived from Crash Cymbals) corresponded to a sharp jagged shape while soft timbres (e.g., sounds derived from Piano) corresponded to a rounded shape. In addition, timbres which had elements of softness and harshness together (e.g., sounds derived from Sax) corresponded to a mixture of previous shapes i.e., a shape with rounded and sharp angles. This is consistent with Kiki–Bouba experiment where subjects mostly chose a jagged shape for Kiki and a rounded shape for Bouba (Köhler, 1947; Ramachandran and Hubbard, 2001; Parise and Spence, 2012). It is also consistent with Parise's findings where subjects associated sine waves (soft sounds) with a rounded shape and square waves with a sharp angular shape (Parise and Spence, 2012).

The correspondence between timbre and shapes was so strong that it had not been affected by color or grayscale. This can be interpreted as a sort of perceptual constancy in audio–visual correspondences. Constancy phenomenon exists in both auditory and visual systems (Erickson, 1975; Walsh and Kulikowski, 1998). Visual constancy examples (Walsh and Kulikowski, 1998) include color constancy, size constancy or shape constancy where the color, size or shape of an object remains relatively constant under different conditions e.g., a red apple looks red at midday or at sunset or TV screen is always seen as a rectangular object regardless of the angle we look at it. An example of constancy in the auditory system is the identification of musical instrument as constant, though timbre, pitch or loudness may change (Erickson, 1975).

Fundamental frequency, on the other hand, played a role when selecting a shape for a given sound. The effect of fundamental frequency on shape selections can be considered as three filters: a band-pass filter passing only the rounded shape associated with soft timbres; a band-stop filter passing only the rounded or mixed shape associated with timbres possessing elements of harshness and softness together; and a high-pass filter passing only the jagged shape associated with harsh timbres. This effect is particularly very important in sensory substitution systems where any visual correlate(s) of timbre should change with fundamental frequency. The claimed synesthetic or audio–visual experiences, music training, age and sex had no effect on timbre-shape associations as DBSCAN found only one group of subjects based on the selected shapes.

Subjects made associations between some timbres and some colors (grayscales). Although these associations are not as strong as timbre shape associations, subjects have associated soft timbres with blue, green or light grayscales and harsh timbres with red or yellow or black. This supports Abbado's ideas in the design of an audio–visual piece of music where he had used keen colors for sharp shapes (e.g., a yellow triangle), and soft, deep colors for round shapes (e.g., a blue circle) (Abbado, 1988). Fundamental frequency had no significant effect on timbre-color associations. This also has to be an important consideration in the design of substitution systems where the color of a timbre should not change significantly with fundamental frequency.

While low and high fundamental frequencies were somewhat associated with dark and light grayscales respectively, results, however, question non-synesthetes' ability to associate fundamental frequency of a complex sound with color or height of a visual shape. This is particularly at odds with previous experiments where subjects had matched height and fundamental frequency (Melara and O'Brien, 1987; Ben-Artzi and Marks, 1995; Patching and Quinlan, 2002; Gallace and Spence, 2006; Rusconi et al., 2006; Eitan and Timmers, 2010; Evans and Treisman, 2010; Chiou and Rich, 2012). The difference, however, may lie in the experimental methodology and auditory stimuli. Simple auditory stimuli have often been used in these experiments. In many cases one auditory feature has been compared with one visual feature e.g., subjects have been asked to judge the correspondence between the height of visual stimulus and the frequency of a simple tone. Such settings are not unbiased because subjects are made to compare only two features (which are assumed to be correlated) and associate them. To circumvent this problem, we used visual stimuli varying in color, gray scale, height and shape and auditory stimuli varying in fundamental frequency and timbre. Our results suggest that, when comparing complex auditory and visual stimuli, subjects may only have considered timbre and shapes rather than height and fundamental frequency.

Only fundamental frequencies within human pitch range were used. Considering the fact that timbre-shape associations were affected by fundamental frequency in this range, the effects of higher fundamental frequencies should be investigated in future experiments. Another useful experiment is to use speech signals (e.g., vowels) as auditory stimuli to study the role of human pitch and formants in the context of auditory–visual correspondences. For instance, the speech sounds have been demonstrated to be involved in the perception of the horizontal or vertical elongation of visual shapes (Sweeny et al., 2012).

Based on the results, sounds can be divided into three categories; those associated with rounded shapes, those associated with jagged shapes and those associated with mixed (rounded and jagged) shapes. We believe that each category of sounds have some features in common which invoke the selection of a particular shape. Similarly, in Guzman-Martinez et al. (2012) a consistent mapping between specific auditory amplitude modulation rates and visual spacial frequencies have been revealed. Signal processing techniques can be used to find the common features. The identification of common features has two advantages: on one hand, it is helpful to find a proper solution for auditory–visual substitution systems and on the other hand it lends itself to the identification of timbre dimensions.

## Author contributions

Mohammad Adeli conceived the experiments, the web site, analyzed the data, and wrote the manuscript. Jean Rouat conceived the idea of study and contributed to the research, the data analyses, and manuscript writing. Stéphane Molotchnikoff contributed to the data analyses.

## Funding

This work was supported by grant ACELP-3IT from Université de Sherbrooke and grant FRQ-NT from Québec government.

### Conflict of interest statement

The authors declare that the research was conducted in the absence of any commercial or financial relationships that could be construed as a potential conflict of interest.
