# Supplementary material for: Audiovisual correspondence between musical timbre and visual shapes
Source: Front Hum Neurosci. 2014 May 30;8:352. doi: 10.3389/fnhum.2014.00352 (PMC4038957; doi:10.3389/fnhum.2014.00352)
Supplement: Supplementary file 1 [file DataSheet1.PDF]

Frontiers in Human Neuroscience

Human Neuroscience
15 April 2014

Supplementary Material: Audiovisual correspondence between musical timbre and visual shapes

Mohammad Adeli [1,*], Jean Rouat [1,2] and Stéphane Molotchnikoff [2,1]

[1] *Neurocomputational and Intelligent Signal Processing Research Group (NECOTIS) Lab, Département de Génie Électrique et de Génie Informatique, Uinversité de Sherbrooke, Sherbrooke , QC, Canada* [2] *Neuroscience Lab, Département de Sciences Biologiques, Université de Montréal, Montreal, QC, Canada*

Correspondence*:
Mohammad Adeli
Neurocomputational and Intelligent Signal Processing Research Group (NECOTIS) Lab, Département de Génie Électrique et de Génie Informatique, Uinversité de Sherbrooke, Boul. de l'Université, Sherbrooke , QC, J1K 2R1, Canada, mohammad.adeli@usherbrooke.ca

1 SUPPLEMENTARY TABLES AND FIGURES

The following table includes the results of all the statistical tests performed to compare 3 age groups. No difference exists between age groups and thus age has not been an important factor in shape selections.

Supplementary Table 1. Comparison of 3 age groups based on shape selections: G1 (19 to 30 years old), G2 (30 to 45 years old) and G3 (46 to 63 years old) represent the age groups. For every two groups, 23 Fisher's exact tests were performed (one test per sound). p-values for a population of 119 subjects are presented in this table. Bonferroni adjusted significance level for each test is 0.00217 . No significant difference was observed between age groups. This implies that age has not been an important factor in shape selections.

	**G1-G2**	**G1-G3**	**G2-G3**
**Sound**	**p-value**	**p-value**	**p-value**
Cello 100	1	.012	.062
Cello 150	.090	.076	.332
Cello 200	.282	.898	.859
Cello 250	.212	.597	.116
Guitar 100	.588	.800	.451
Guitar 150	.469	.500	.154
Guitar 200	.183	.302	.111
Guitar 250	.663	.172	.445
Piano 100	.838	.103	.085
Piano 150	1.000	.335	.270
Piano 200	.302	.013	.178
Piano 250	.157	.463	.390
Marimba 100	.396	1.000	.622
Marimba 150	.329	.372	.762
Marimba 200	.345	.042	.332
Marimba 250	1.000	.024	.029
Sax 100	.897	.392	.679
Sax 150	.737	.295	.309
Sax 200	.901	.108	.110
Sax 250	.035	.152	.004
Triangle	1.000	1.000	1.000
Crash Cymbals	.679	.554	.740
Gong	.776	.630	.460
